# Nanoscale Protein Diffusion by STED-Based Pair Correlation Analysis

**DOI:** 10.1371/journal.pone.0099619

**Published:** 2014-06-26

**Authors:** Paolo Bianchini, Francesco Cardarelli, Mariagrazia Di Luca, Alberto Diaspro, Ranieri Bizzarri

**Affiliations:** 1 Nanophysics, IIT—Italian Institute of Technology, Genoa, Italy; 2 Center for Nanotechnology Innovation @NEST, Istituto Italiano di Tecnologia, Pisa, Italy; 3 NEST, Scuola Normale Superiore and Istituto Nanoscienze - CNR, Pisa, Italy; 4 Istituto di Biofisica – CNR, Pisa, Italy; University of Sydney, Australia

## Abstract

We describe for the first time the combination between cross-pair correlation function analysis (pair correlation analysis or pCF) and stimulated emission depletion (STED) to obtain diffusion maps at spatial resolution below the optical diffraction limit (super-resolution). Our approach was tested in systems characterized by high and low signal to noise ratio, i.e. Capsid Like Particles (CLPs) bearing several (>100) active fluorescent proteins and monomeric fluorescent proteins transiently expressed in living Chinese Hamster Ovary cells, respectively. The latter system represents the usual condition encountered in living cell studies on fluorescent protein chimeras. Spatial resolution of STED-pCF was found to be about 110 nm, with a more than twofold improvement over conventional confocal acquisition. We successfully applied our method to highlight how the proximity to nuclear envelope affects the mobility features of proteins actively imported into the nucleus in living cells. Remarkably, STED-pCF unveiled the existence of local barriers to diffusion as well as the presence of a slow component at distances up to 500–700 nm from either sides of nuclear envelope. The mobility of this component is similar to that previously described for transport complexes. Remarkably, all these features were invisible in conventional confocal mode.

## Introduction

The ability of fluorescence microscopy to probe intracellular processes led to the development of powerful approaches to unveil the diffusive dynamics of biomolecules. Among these, Fluorescence Correlation Spectroscopy (FCS) established as an efficient way to obtain single-molecule diffusion with high statistics in unperturbed cells [1]. In FCS, the diffusion dynamics are recovered from the fluorescence fluctuations generated by single molecules that cross the focal volume (defined by the point spread function, or PSF) during their motion [2]. Traditionally, FCS is carried out as a single point measurement (spFCS). Yet, spFCS lacks completely of spatial information: local diffusivity is assessed one point at a time with no details about the routes the molecules take prior to crossing the PSF. Vice-versa, in spatiotemporal FCS (stFCS) [3], the PSF is moved in a periodic pattern within the sample at a rate faster than diffusion fluctuation, thus introducing a spatial component into the measurements. Different stFCS approaches stem from the several possible spatial patterns of the moving PSF [2]. Repeated PSF scanning over a circle or a line is particularly useful as: 1) it is equivalent of performing many spFCS measurements simultaneously, yielding a spatial map of molecular diffusivity [4], 2) it allows cross (“pair”) correlation (pCF) between any two points in the line separated by *n* pixel [pCF(*n*)], “mapping” the routes taken by molecules from the first to the second observation point [5], [6].

The sensitivity of pCF to detect even subtle dynamic processes critically relies on the spatial resolution of the microscope being used. Confocal microscopes allow a radial resolution that is limited in the 200–250 nm range. The recent practical advent of the RESOLFT “concept” demonstrated how such a limitation can be circumvented in fluorescence microscopy without infringing any physical law [7]. The most striking example is represented by STED imaging: by superimposing an excitation and a depletion PSF of different geometry and scanning them over the sample, super-resolution close to 20 nm in biological specimen was demonstrated [8]. Depletion of the excited state is carried out by either a continuous wave (CW) or a pulsed laser beam whose wavelength falls on the red tail of the emission spectrum of the probe under examination. STED was shown to be applicable to several fluorophores, including widely used autofluorescent proteins (FPs) such as EGFP (Enhanced Green Fluorescent Protein) [9] and YFP (Yellow Fluorescent Protein) [10]. From a biological perspective FPs are particularly relevant as they allows for genetic label of almost any intracellular protein [11]. Both EGFP and YFP can be effectively depleted at wavelengths slightly below 600 nm [9], and a STED/confocal system supplied with a 592 nm CW depletion laser source has become recently available on the market.

STED was recently applied to spFCS to study membrane lipid dynamics in PSF of variable size down to 30 nm [12], [13]. Yet STED is *a raster scanning imaging technique*, being perfectly amenable to the stFCS approach [14]. It is surprising, however, that no reports on STED applied to pCF has been reported yet. In this work we shall prove that pCF and STED imaging can be proficiently combined (STED-pCF) to obtain bio-molecular diffusion maps at a spatial resolution beyond the one allowed by diffraction limit. Notably, we applied STED-pCF to genetically-encoded constructs of EGFP or YFP in the commercial CW-STED imaging setup, to demonstrate the straightforward applicability of our approach.

## Materials and Methods

### Materials

All inorganic and organic materials were purchased from Sigma-Aldrich.

### Molecular and Cell Biology Methods

#### Expression and purification of HBcAg-YFP capside-like particles (YFP-CLPs)

A plasmid pET28a encoding internal fusions of the HBV core protein (HBcAg) with monomeric YFP [15] were kindly provided by Dr. Michael Nassal (University Hospital Freiburg). The plasmid was propagated and maintained in *E. coli* Top10 cells (Invitrogen), and was transformed into BL21(DE3) *E. coli* (Invitrogen) for expression as recommended by the producer.

For the YFP-CLPs expression, BL21 *E. coli* transformants were plated on LB agar medium containing 50 µg/ml of ampicillin and a single colony were picked for overnight culture in 5 mL of LB medium containing ampicillin (50 µg/ml). Then the overnight cultures were used to inoculate fresh LB medium with appropriate antibiotic for the selection to an OD_600_ of 0.05–0.1 (∼1∶50 dilution of the overnight culture) on a shaking platform. The cultures were grown until an optical density at 600 nm ∼0.4, then induced by IPTG (0.5 mM) and, finally, were further incubated for an additional 16 h at room temperature (20° to 22°C). Bacteria were harvested by centrifugation (6,000 g, 15 min.), and the cell pellets were washed using TN-50 buffer (25 mM Tris/HCl, pH 7.5; 50 mM NaCl). For lysis, the frozen cells were suspended in TN-50 buffer (5 ml for the cells from a 250 ml culture) adding protease inhibitor (Roche), incubated with 40 mg lysozyme and incubate on ice for 30 minutes. Then, the solution was sonicated on ice using six 30-second bursts at high intensity with a 60-second cooling between each burst. The raw lysate was further drawn through a syringe needle several times. Cellular debris and insoluble proteins were separated by 2-times centrifugation (6,000 g, 30 min at 4°C) and the supernatant was further filtered by 0.22 µm Millipore syringe filter. Three milliliters of cleared supernatant were directly subjected to sedimentation in 10% to 60% (w/v) sucrose step gradients (3 ml of 10% and 5-ml each of 20%, 30%, 40%, 50%, and 60% sucrose) using a F0630 30° Fixed-Angle Rotor by Beckman (57,000 g, 4,5 h, 20°C). Under these conditions, the YFP-CLPs were recovered between 40% and 50% sucrose fractions of the gradient. Fractions containing the YFP-CLPs were pulled, dialyzed against TN-300 buffer (25 mM Tris/HCl, pH 7.5; 300 mM NaCl) and concentred by ultrafiltration (Amicon Ultra devices, 100 KDa, cut-off; Millipore).

#### Preparation of plasmid encoding for NLS-GFP construct

The plasmid codifying for EGFP fused at the *N*-terminus to the C-terminus of Simian Virus 40 Nuclear Localization Signal (NLS: MYPKKKRKVEDP) was engineered as reported in [16]. Shortly, NLS-GFP plasmid was constructed by two rounds of multiple point mutations onto the sequence of MYGRKKRRQRRR-EGFP (denoted as Tat11-EGFP) pcDNA3.0 plasmid (Invitrogen, Carlsbad, CA) using the QuickChange Kit (Stratagene). The two primers used in the mutagenesis reactions to transform the Tat11 amino acid sequence in the NLS of SV40 were 5′-GGATCCATGTATCCCAAGAAGAAGCGGAAAGTGCGACGAAGA-3′ and 5′-GAAGCGGAAAGTGGAAGACCCAAAGCTTATAGTGAGC-3′. Antisense primers had reverse complementary sequences.

#### Cell culture and transfection

CHO-K1 cells were grown in Ham's F12K medium supplemented with 10% of fetal Bovine Serum at 37°C and in 5% CO_2_. CHO-K1 cells were plated on imaging dishes and transiently transfected using Lipofectamine 2000 according to manufacturer's protocol. The plasmid encoding for NLS-GFP used here has been described in detail in ref. [16].

### Fluorescence imaging methods

#### Confocal/STED microscopy setup

Measurements were performed by means of a Leica TCS SP5 STED (Leica-microsystems, Mannheim, Germany) inverted confocal/STED microscope. Excitation was provided by 488 nm Ar laser line and detection was done in the 500–550 nm range (Semrock filter) by one avalanche photodiode detector. Pinhole was set to 0.6–1 Airy size. Line scanning speed ranged from 10 to 1400 Hz in standard acquisition mode, whereas it was set to 8000 Hz in fast acquisition mode by using the resonant scanner system of the microscope. In STED mode, the 592 nm CW laser beam was superimposed at a typical power of 200–300 mW before the objective.

#### Preparation of a polyacrylamide gels gel entrapping YFP-CLPs

Five microlitres of YFP-CLPs solution were spotted on an agarose gel (1% w/v) and allowed to dry and penetrate into gel matrix. For imaging experiments a slice containing YFP-CLPs were cut and put face down on glass-bottom Petri dishes (Wilco Wells).

#### Confocal/STED analysis of YFP-CLPs in polyacrylamide gels

**Figure 1:**
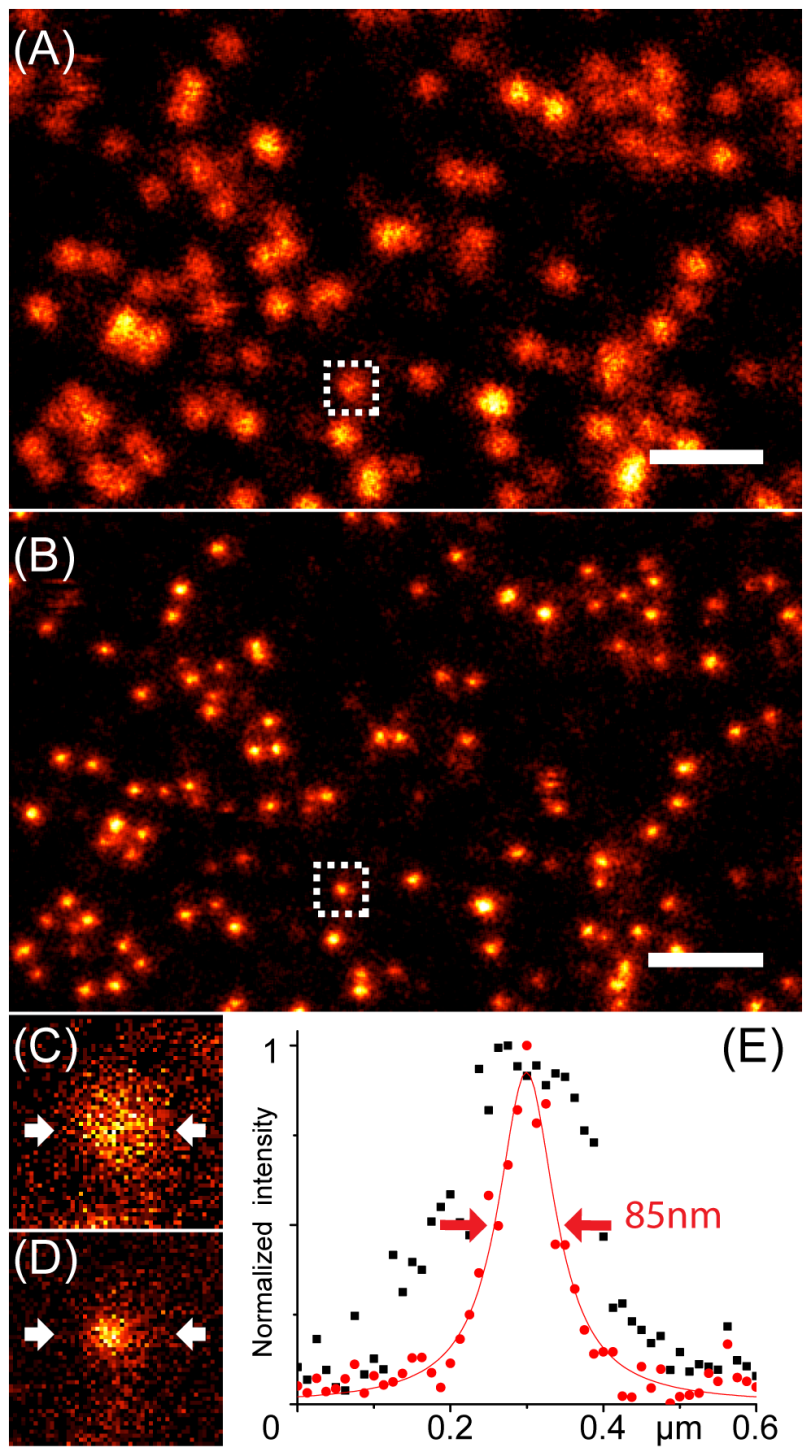
Confocal (A) and STED (B) images of YFP-CLPs entrapped in acrylamide gel. Scale bar: 1 µm. Zoom on the single YFP-CLP enclosed in the white dotted square is reported in panel C (confocal) and (D) STED. Plot (E) shows the pixel intensity distribution along a horizontal line crossing the single YFP-CLP of images (C) and (D) and passing through its center; the intensity *vs.* distance distributions of confocal and STED acquisitions were fitted to Gaussian curves and the calculated Full Width at Half Maximum values were taken as spatial resolutions of the two methods.

CLP-YFPs entrapped in polyacrylamide gel were imaged by acquiring xy images in confocal or STED mode. Acquisition parameters were: 2048×2048 pixels, 1400 Hz line scanning speed, 64 line averages. The radial (xy) dimension of selected YFP-CLPs (such as the one reported in Fig. 1C–D) was determined by plotting the pixel fluorescence intensity collected along an horizontal line crossing the target CLP particle and passing through its center (see Fig. 1E). The intensity *vs.* distance distribution was fitted to a Gaussian curve and the Full Width at Half Maximum (FWHM) was taken as spatial resolution of the image (*w_xy_*).

#### Confocal/STED line-scan measurements (xt acquisitions) and pCF

In confocal (depletion laser off) or STED (depletion laser on) we acquired data by raster scanning the excitation (and, in STED mode, the depletion) laser beam(s) along a 64-pixel line (1 pixel = 50 nm). Raster scanning along the same line was repeated >2·10^5^ times (*xt* acquisition), in fast acquisition mode (8000 Hz = 0.125 ms/line). The *xt* acquisition generated an “intensity carpet” made of >2·10^5^ rows and 64 columns, where the *x* and *y* coordinates represent space and time, respectively.

*xt* acquisition were carried out on YFP-CLPs in buffer (20 mM diethanolamine, pH 8–8.5) or in CHO cells transiently expressing EGFP or NLS-GFP. In cells expressing NLS-GFP, the scanned line was set to cross perpendicularly the interface between cytoplasm and nucleus. The actual position of the nuclear envelope along the line was determined by a MATLAB (R2010a, The MathWorks, Natick, Massachusetts, USA) routine. The routine reads the Leica proprietary file by the use of the LOCI bioformat library [17]. For each experiment we reduced the number of lines from 204,900 to 10,245 by averaging every 20 lines. On account of the uneven distribution of fluorescence between nucleus and cytoplasm, the fluorescence profile (*I(x) vs. x*, *x* expressed in pixels) of each line was fitted by the following sigmoid function:
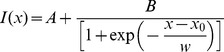
(1)


The result of such an analysis was a distribution of values for each of the free parameters in the equation (*A*, *B*, *x_0_*, *w*). In our analysis *x_0_* and *w* represented the midpoint and width of NE barrier. Finally each distribution was fitted to a Gaussian function to recover the average and standard deviation of *x_0_* and *w* for each cell.

For all *xt* acquisition and relevant intensity carpets, we calculated the pair-correlation functions (pCF) on the intensity carpet by the SimFCS software (Laboratory for Fluorescence Dynamics). Average changes of fluorescence intensity over time in the sample (photobleaching, cell movement) were filtered out by adding random uncorrelated counts, following the detrend approach reported in ref. [18]. Documentation for the SimFCS software can be found at www.lfd.uci.edu. Further details can be found also in ref. [5].

#### Determination of diffusion coefficient or optical resolution from pCF(0)

The diffusion coefficient of freely diffusing CLP-YFPs in buffer (diethanolamine 20 mM, pH 8) was determined by fitting the confocal-pCF(0) to the analytical autocorrelation function for 3D isotropic diffusion [1]:
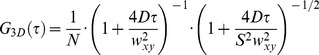
(2)In eq. 2, *N* is the number of diffusing particles in the confocal volume, *D* is the diffusion coefficient, 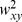 is the optical resolution (actually the radial size of Point Spread Function, PSF), and *S* is the ratio between the axial and radial size of PSF. For fitting, we fixed *w_xy_* = 275 nm and *S* = 5, whereas *D* and *N* were left to vary.

Vice-versa, the optical resolution was obtained from the STED-pCF(0) of freely diffusing EGFP in CHO nucleus by fixing *D* = 20 µm^2^/s [16] and *S* = 10 in eq. 2, and letting *N* and *w_xy_* free to vary.

#### Analysis of pCF curves and maxima

The mathematical analysis of cross-correlation between fluorescence fluctuations occurring in two spatially-separated focal volumes has been presented originally by Eigen [19], and later by Langoswski [20] and Schwille [21]. Assuming that the two focal volumes are not displaced axially each other, and that the radial distance between their centers is *d*, the cross-correlation function is given by:
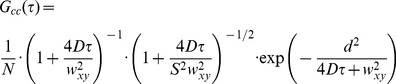
(3)


Eq. 3 can be considered as representing the pair correlation function (pCF) for unrestricted (i.e. in absence of obstacles and barriers) free diffusion of particles. Accordingly, Eq. 3 was used to analyze the 3D free diffusion of CLP-YFP particles in buffer solution. In more details, STED-pCF(3) *vs.* time plots were fitted to eq. 3 by setting *d* = 150 nm (1 pixel = 50 nm) and the axial resolution 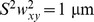 to recover the STED radial resolution 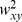 as fitting parameter.

The unrestricted dynamic behavior expressed by eq. 3 seems rather unrealistic in a crowded cell environment characterized by barriers and corrals. Yet eq. 3 was used as a general reference to estimate the local diffusion coefficient of NLS-GFP in cells. It is rather easy to demonstrate that eq. 3 admits a maximum for 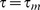 where:
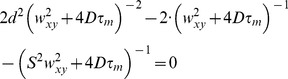
(4)


Eq. 4 can be further simplified assuming that the axial to radial resolution ratio *S* is very large (>5) as obtained in STED (or when diffusion is confined in a 2D space such as the plasma membrane). We have:
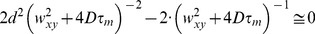
(5)which can be recast as:
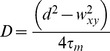
(6)In our STED experiments, 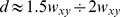, and eq. 7 was further approximated as:
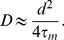
(7)


Eq. 7 was used to estimate the diffusion coefficient of NLS-GFP, either in free form or slowed by binding to some component of import machinery, from the *τ*_m_ value where the STED-pCF(4) carpet displayed the intensity maxima, and by setting *d* = 200 nm (4 pixel).

## Results and Discussion

### STED imaging resolution test on fluorescent Capsid Like Particles (YFP-CLPs)

We initially tested the gain in spatial resolution by STED over confocal imaging in our setup using immobilized YFP-labeled Capsid Like Particles (CLPs) as benchmark. YFP-CLPs were obtained from HBV C149 protein genetically fused with EYFP and a single particle has about 20 nm radius [22], [23]. Agarose gel-immobilized YFP-CLPs appeared as bright fluorescent spots whose diameter was about 200 nm in confocal mode, and 85 nm in STED mode (Fig. 1). We note that, in principle, a single CLP should bear as many as 240 YFP molecules, although less than 50% are thought to be properly folded [22].

#### The pair correlation function (pCF): general description

**Figure 2:**
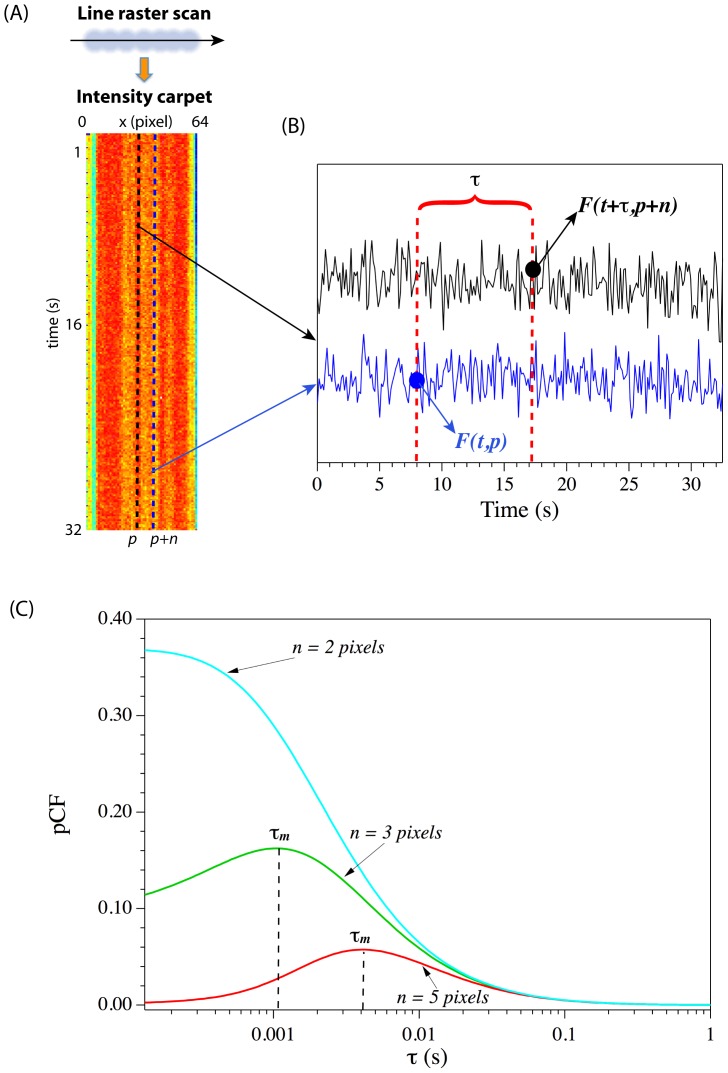
Calculation and characteristics of pCF. (A) From the repeated raster scan of a 64-pixel line (*xt* acquisition), an “intensity carpet” is generated in which the *x*-coordinate represents the pixel position along the line, the *y-*coordinate corresponds to the time of acquisition, and the actual intensity is expressed by a black-to-red color scale. (B) Fluorescence intensity *vs.* time along two generic columns of the carpet separated by *n* pixels. The pCF relevant to the two columns is obtained by considering a variable lag-time 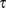 and then applying equation 8 in main text. (C) Simulated pCF *vs.* 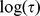 for optically indistinguishable (blue trace, pixel distance *n = 2*) and distinguishable (green and red traces, pixel distance *n = 3* and *n = 5*) locations (columns) along the raster-scanned line.

In all our experiments, pCF was calculated from line-scanning measurements (*xt* acquisitions) in a given region of the sample. In each *xt* acquisition a 64-pixel line (1 pixel = 50 nm) was raster-scanned at 125 µs/line rate (8 kHz) for 2–6·10^5^ times. Fluorescence intensity data were recast under the form of a “carpet” in which the *x*-coordinate represents the pixel position along the line, the *y-*coordinate corresponds to the time of acquisition, and the actual intensity is expressed by a black-to-red color scale (Fig. 2A).

As originally conceived [24], the pCF corresponds to the cross-correlation between the intensity fluctuations occurring along two given columns (i.e. two sample points separated by a given distance) of the *xt* intensity carpet. More formally, the general pCF is obtained from the point intensities *F(t,p)* and *F(t,p+n)*, respectively calculated along the (*p*) and (*p+n*) columns of the carpet (Fig. 2A), as follows:
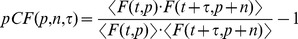
(8)where brackets indicate temporal averaging. In eq. 8 *τ* is the variable time-lag at which the fluctuation events in the *p* and *p+n* pixels of the line are cross-correlated (Fig. 2B).

Positive pCF indicates that some fluorescent particles indeed take a time *τ* to travel from *p* to *p+n*, whereas zero pCF witnesses no communication between the two loci. In general, the 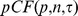 *vs.* 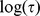 plot may assume either one of these three form [24]:

- A sigmoidal curve with maximum at the shortest sampled *τ* and reaching zero for 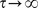 (Fig. 2B, blue trace); this behavior occurs when the pixel distance *n* is contained within the optical resolution of the imaging system, i.e. the points *p* and *p+n* are not optically distinguishable. As special case, for *n* = 0 we have the autocorrelation function (ACF) at the point *p*.
- A bell-shaped curve with maximum at time *τ_m_* (Fig. 2D, green and red traces); this behavior occurs when pixel *p* and *p+n* are optically distinguishable, and *τ_m_* represents the time required by the larger number of particle to travel between the two points. Typically, 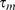 increases with *n* (Fig. 2D, compare *n = 3* and *n = 5*), although the presence of obstacles can produce large 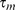 even for short *n* values. Increasing *n* also multiplies the number of alternative pathways connecting the two pixels, resulting in the amplitude decrease and broadening of the pCF (Fig. 2D, compare *n = 3* and *n = 5*).
- A zero pCF at any lag-time; this behavior occurs when a barrier prevents any diffusion between the two pixels.

When unrestricted random walk is the only diffusion modality of the fluorescent particle, eq. 3 (Materials and Methods) describes formally behaviors (a) and (b). Yet this special regime is rather unrealistic in the cell context where obstacles, barriers, and anomalous diffusion are extremely frequent [24], [25].

For a given *n* value, pCF is usually presented as a 2D carpet (pCF carpet) in which the *x*-coordinate represents the pixel position along the line, the *y-*coordinate corresponds to the lag-time, and the pCF amplitude is expressed by a black-to-red color scale. In some cases, spatial averaging of pCF over some or all *p* pixels of the line contributes to reduce noise while still affording relevant information on the particle dynamics. Regardless of the presentation mode (carpet or spatially-averaged curve), the pair correlation is usually denoted in a short form as pCF(*n*).

#### STED-pCF resolution test on freely-diffusing YFP-CLPs

**Figure 3:**
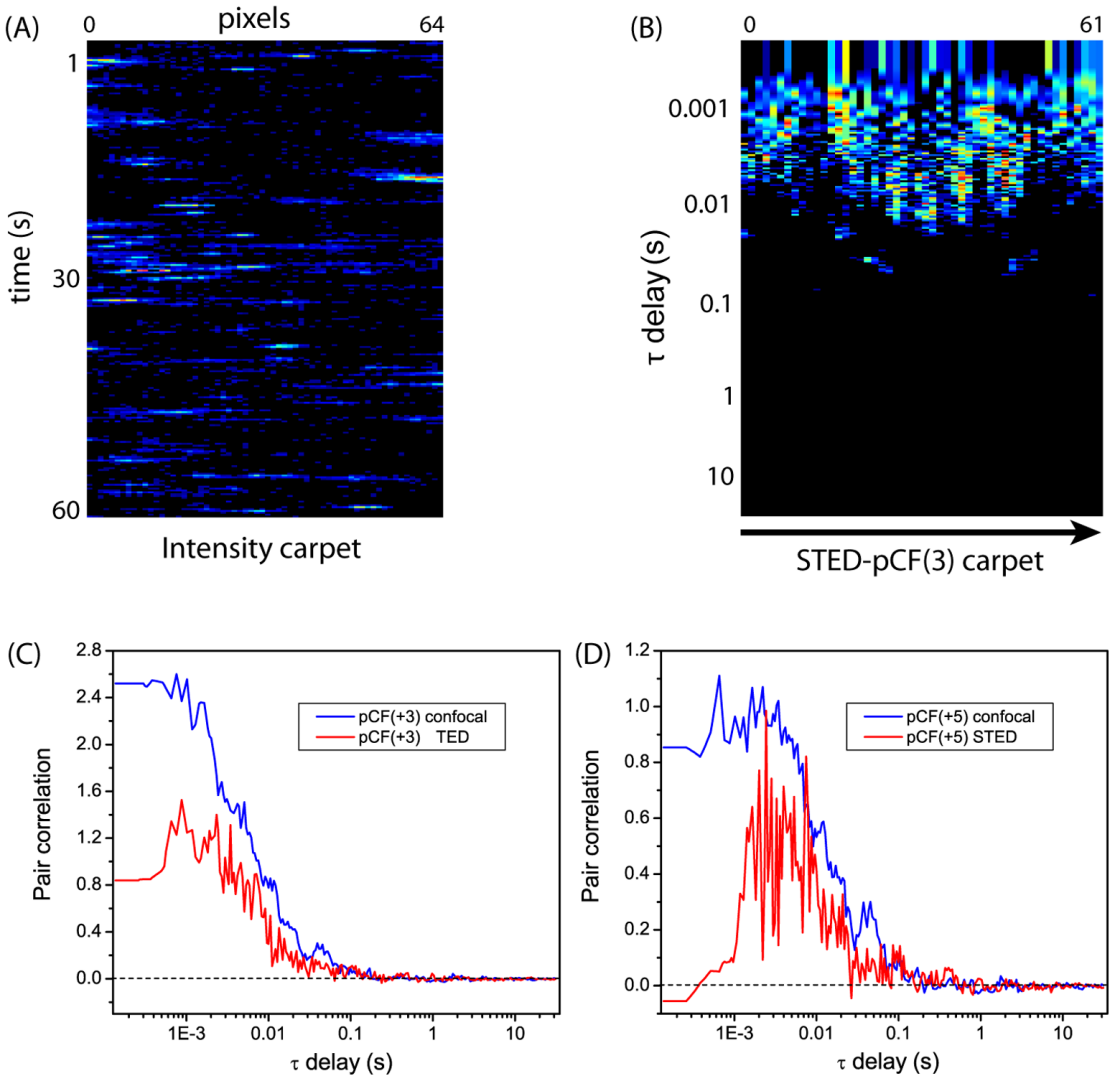
pCF analysis of diffusing CLP-YFP. (A) Intensity and (B) pCF(3) carpets for free diffusion of CLP-YFPs. Comparison between the averaged (on 64 pixels) confocal (blue) and STED (red) pCF curves is reported for pCF(3) (C) and pCF(5) (D).

The diffusion of freely-diffusing YFP-CLPs in buffer was analyzed by pCF either in confocal or STED mode. In both cases, the intensity carpets highlighted a few, transient, YFP-CLPs crossings of the scanned line, as expected for single capsid regime (Fig. 3A). Fig. 3B represents a typical STED-pCF(3) carpet, whereas Fig. 3C shows the comparison between the average STED (red) and confocal (blue) pCF(3) traces. STED-pCF(3) has a bell-shaped form, whereas confocal-pCF(3) has a sigmoidal form. Following the general pCF description reported in the previous section, we can conclude that a 3-pixel distance was optically-distinguishable in STED but not in confocal mode. As expected, at longer distances (5 pixel, 250 nm), STED-pCF showed a time-delayed correlation maximum compared to STED-pCF(3) (Fig. 3C–D, red traces); yet confocal-pCF(5) could not resolve the two spatial positions yet (Fig. 3D, blue trace). Although CLP-YFPs can not be truly considered as “point” particles with respect to the PSF size, we estimated the value of the STED resolution by means of eq. 3 (Materials and Methods), which represents the cross-correlation function for fluctuations collected in two focal volumes radially-displaced by a given distance *d* and produced by freely-diffusing “point” particles [19]–[21]. We found out a STED resolution of 106±17 nm (average of 7 carpets). This value is somewhat larger than observed for immobile CLP-YFPs. Nonetheless, we should stress that the observed resolution represents a very good figure for a CW-STED system, as this configuration trade off instrumental simplicity with poorer fluorescence contrast along the STED-PSF slope, and it is thus characterized by worse resolution as compared to pulsed-STED [26].

Finally, fitting confocal-pCF(0) (confocal-ACF) to an isotropic 3D diffusion model yielded *D* = 8.5±2.5 µm^2^/s, a value very consistent with that calculable for rigid spheres having 20 nm radius at RT in water. In this calculation, we assumed a confocal PSF size (*w_xy_*) of 275 nm, as determined by ACF measurements on single GFP (see next section). Our results indicate that CW-STED improved more than 2.5 fold the imaging resolution of moving particles as compared to confocal mode.

#### Test measurements on EGFP in living cells

STED is always associated to a reduction of collected fluorescence, on account of the depletion mechanism of the excited state and, often, by other photophysical processes induced by the depletion source (e.g. absorption to higher excited states and subsequent bleaching/conversion to triplet [27]). Fluorescence reduction is always associated to a degradation of signal to noise ratio (S/N). YFP-CLPs are extremely bright systems and they afforded a large S/N ratio. In most cell imaging experiments, however, only 1–2 fluorescent reporters per target biomolecule are into play and much lower S/N ratios are achievable. Furthermore, the closed volume of a cells raises the issue of photobleaching, as the molecules are bounded to cross several times the PSF. In order to model a typical experiment, we set out to determine the resolution of our STED-pCF approach on monomeric EGFP transiently expressed into cultured Chinese Hamster Ovary cells (CHO). Line scanning measurements were performed as for CLPs in both STED and confocal mode along a 64-pixel line within the cell nucleoplasm. Then, confocal and STED-pCF(0) traces were fitted to isotropic 3D diffusion model equation setting *D* = 20 µm^2^/s (diffusion coefficient of a monomeric GFP in the nucleoplasm [28]) to recover the PSF size *w_xy_*. Although the cell nucleoplasm cannot be considered a spatially-infinite solution, because it is bounded by the nuclear membrane, the use of the 3D isotropic model is justified as long as the radial and axial PSF dimensions are much shorter than the nuclear axes. In CHO cells, nuclear axes range from 10 to 20 µm, i.e. at least 10-fold larger than the axial size of PSF 0.8–1.0 µm.

**Figure 4:**
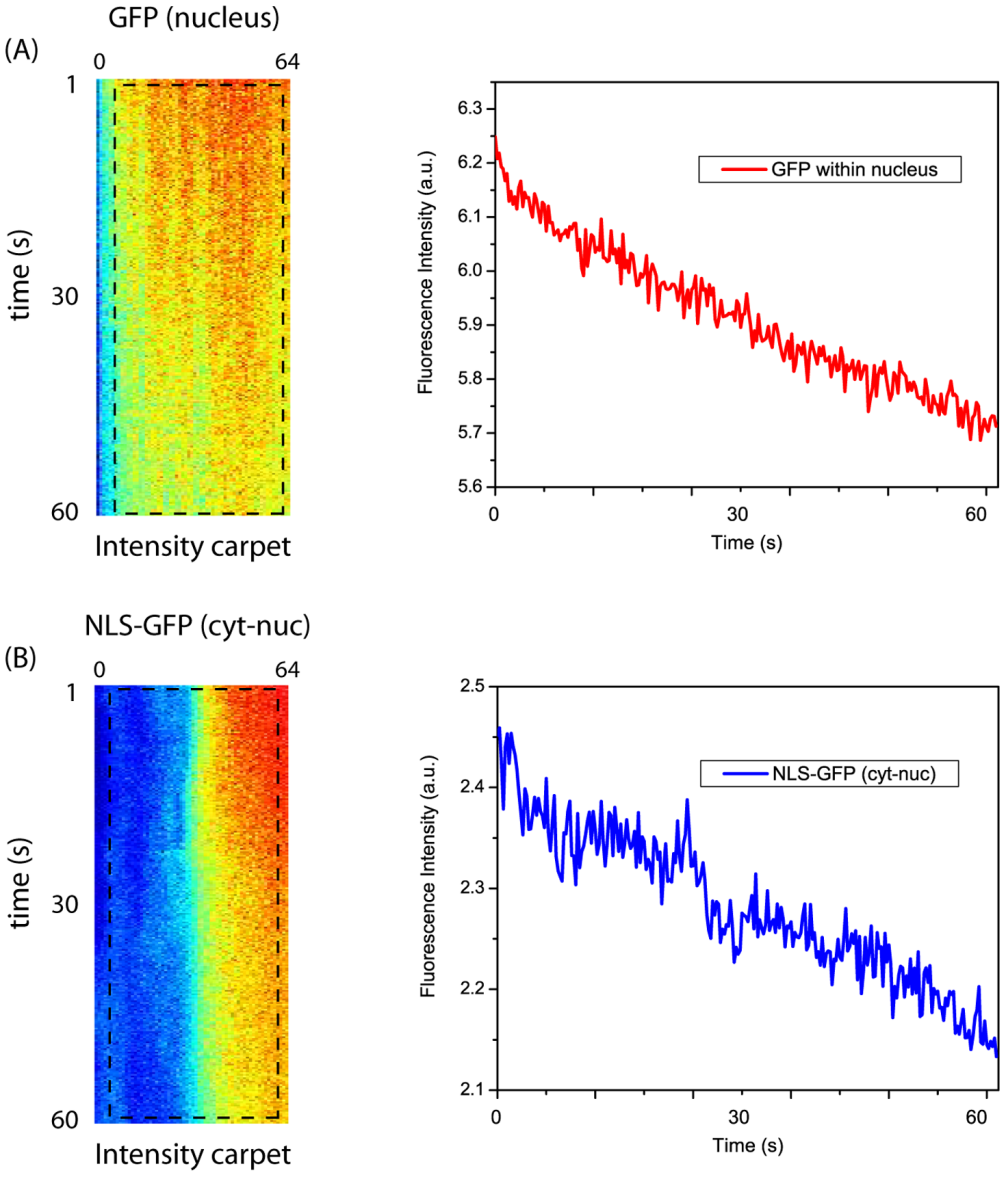
Typical photobleaching of EGFP (A) and NLS-EGFP (B) in STED *xt* acquisition. On the left of each panel, the intensity carpet is displayed; on the right it is shown the plot of the average intensity *vs.* time as determined in the dotted rectangle visible in the carpet.

We found *w_xy_* = 110±25 nm for STED-pCF and *w_xy_* = 275±8 nm for Confocal-PCF (N = 8 cells). These findings confirm that STED mode provided about 2.5-fold resolution improvement over confocal mode even for measurements carried out on a single GFP, i.e. on a particle for which the S/N ratio is much lower than for CLP-YFPs (each CLP-YFP contains 240 YFPs). We should note that EGFP photobleaching under STED condition accounted to about 5% in 60 s acquisition (∼5·10^5^ lines, Fig. 4A) and was easily accounted for by the detrend procedure of pCF calculation (see Materials and Methods) or, alternatively, by calculating pCF on a 1.5–2·10^5^-lines subset of the intensity carpet.

#### Measurements of protein cargo actively imported into the cell nucleus

Compared to conventional confocal mode, STED-pCF appears particularly promising to test diffusion profiles in the close proximity of subcellular compartments where phenomena such as binding and mobility restriction may play a relevant role. Accordingly, we set out to test how the proximity to nuclear envelope affects the mobility features of molecules actively imported into the nucleus. As fluorescent reporter, we selected EGFP fused to the Nuclear Localization Sequence MYPKKKRKVEDP of Simian Virus 40 (NLS_SV40_). NLS_SV40_ is a powerful promoter of active protein transport from the cytoplasm towards the nucleus. Indeed, NLS_SV40_ contains a single stretch of basic residues that binds with micromolar affinity to the protein transporter complex Importin-alpha∶Importin-beta in the cytoplasm [29]. The full complex is subsequently relocated into the nucleus through facilitated transport across the NPC. The complex is disassembled in the nucleus by the action of RanGTP (whose nuclear accumulation is a function of metabolic energy), with release of NLS_SV40_. Therefore, NLS_SV40_ is capable of transferring to the nucleus any linked protein cargo whose molecular weight is above the limit (40–60 kDa) for passive (i.e. dependent on concentration gradients) diffusion through the NPC [28]. In this context, EGFP represents a particular cargo for NLS_SV40_, as the rather low molecular weight of NLS_SV40_-EGFP (27 kDa) allows for concomitant active and passive shuttling across the NPC. Accordingly, actively transported NLS_SV40_-EGFP in the nucleus is then recycled back to cytoplasm by passive diffusion, establishing a steady-state condition that maintains significant molecular fluxes across the NPC at any time of the non-dividing cell [30]. This makes NLS_SV40_-EGFP (hereafter denoted simply as NLS-GFP) particularly suitable to study active nuclear import, as we previously demonstrated by both FRAP [16], [28], [30] and confocal pCF [5].

**Figure 5:**
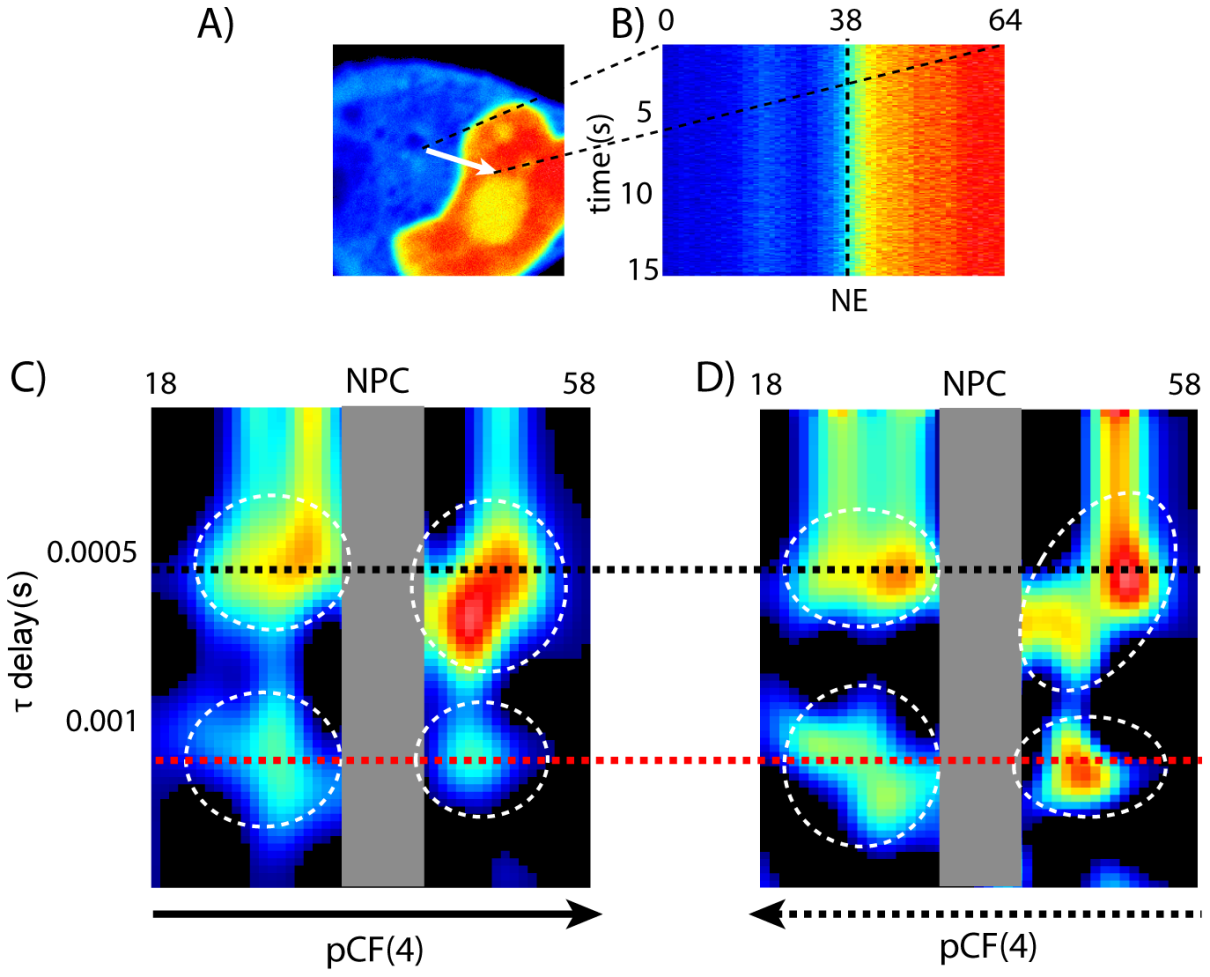
STED-pCF analysis of NLS-GFP in cells. (A) Fluorescence intensity image of a cell expressing NLS-GFP: the nuclear accumulation is clearly visible. (B) Fluorescence intensity carpet in which the *x*-coordinate corresponds to the pixels along the scanned line (denoted as an arrow in A) and the *y*-coordinate corresponds to the time of acquisition (seconds). pCF(4) carpets obtained along the N→C (C) and C→N (D) directions.

NLS-GFP partitions preferentially in the nucleus (Fig. 5A), and the interface between nuclear and cytoplasmic fluorescence (hereafter denoted as N/C) is clearly distinguishable (Fig. 5B). Line scanning measurements were performed in both STED and confocal mode along a 64-pixel line crossing the nuclear envelope (NE) (Fig. 5A). We were able to collect up to about 5.0×10^5^ scan lines (∼60 s) with maximum 10–12% photobleaching of the nuclear region in STED mode (Fig. 4B). Detrend procedure (see Materials and methods) or analysis of a subset of the intensity carpet were applied to avoid photobleaching effects as well as other intensity drifts (e.g. movement of the NE barrier) on pCF calculation.

**Figure 6:**
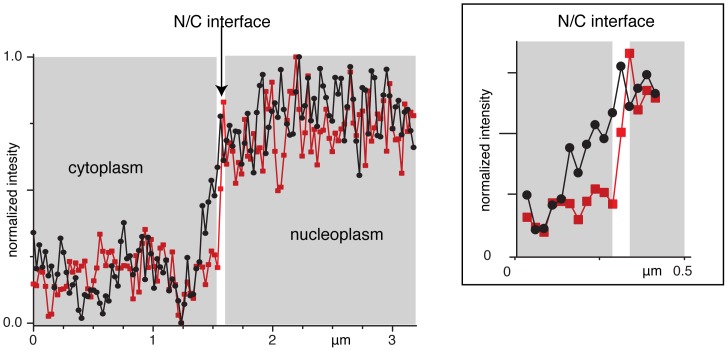
Sigmoidal intensity profile along a line crossing the nuclear envelope from cytoplasm (0–1.5 mm) to as imaged in confocal (red) and STED (black) modes. Note the larger width of N/C interface detected in confocal mode (4–6 pixels) as compared to STED (2–3 pixels) mode.

Before carrying out the STED-pCF analysis, we identified the position and width of the N/C by fitting the fluorescence profile along the scanning line to a sigmoidal curve (see Materials and Methods section for further details). Note that the measured N/C width stems from the convolution of the microscope PSF and actual N/C size. The latter should include the inner and outer nuclear membranes as well as the nuclear lamina, with an estimated overall thickness of 50–100 nm [31]. As expected, smaller width of N/C was found in STED (2–3 pixels, 100–150 nm) as compared to confocal mode (5–6 pixels, 250–300 nm nm) (Fig. 6).

**Figure 7:**
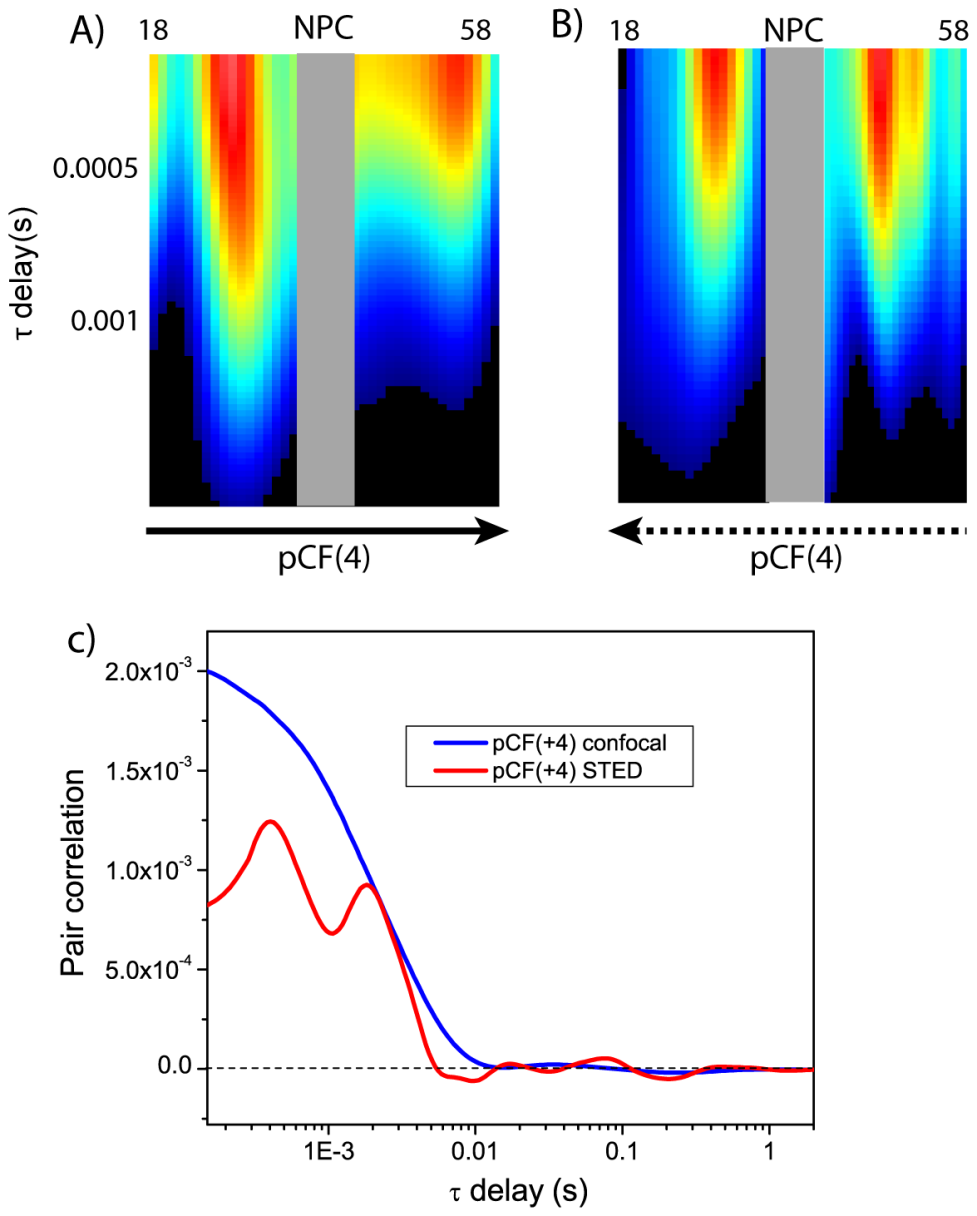
Confocal-pCF analysis of NLS-GFP in cells. (A,B) Confocal-pCF(4) carpets obtained along the N→C (A) and C→N (B) directions along a line crossing the N/C interface. The two carpets are the confocal-pCF analogs of the STED-pCF carpets reported as Figure 3C,D in main text. (C) Confocal (blue) and STED-pCF(4) (red) curves for pixel 28 in the scanned line across the nuclear envelope.

We found that STED-pCF solved well a 4-pixel distance while confocal mode could not (Fig. 5D,C and Fig. 7), suggesting a resolution in the 100–150 nm range, in keeping with STED-pCF data on monomeric EGFP expressed in cell. STED-pCF(4) carpets were subsequently analyzed excluding the closest 4 pixels (i.e. 200 nm) on both sides of the N/C center, a region where NLS-GFP dynamics is actively influenced by nuclear pore components [32]. Notably, STED-pCF(4) carpets revealed a rich NLS-GFP dynamics characterized by one or two pCF maxima, while these features were totally hidden in confocal mode (compare Fig. 5C,D with Fig. 7A). Interestingly, pCF intensity maxima were surrounded by “darker” regions in STED-carpets, indicating the presence of barriers to mobility [5]. These barriers might be due to the structural organization of chromatin and endoplasmic reticulum on the nuclear and cytoplasmic sides, respectively, of the NE [25].

To provide quantitative biological insights from the collected data, for each STED carpet we analyzed the times relevant to pCF maxima (*τ_m_*) by the approximated eq. 7 (Materials and Methods). We found that, in both N and C compartments, a fraction of protein moves similarly to a single free GFP [5] (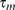 = 0.6±0.3 ms, *D* = 17±8 µm^2^/s, 8 carpets), irrespective of the chosen direction (N→C or C→N) for calculating pCF (Fig. 3C, black dotted line). Yet an additional pCF maxima was always detected at a later times (Fig. 5C,D, red dotted line), indicating an NLS-GFP pools whose diffusion is slowed down by some biological factor. In all carpets, the slow NLS-GFP pool appeared on both the cytoplasmic side (moving C→N) and the nuclear side (moving N→C). Although other factors might be invoked, the simplest hypothesis ascribes the slower diffusion characteristic of this pool to specific binding to intracellular components/proteins. As support to our hypothesis, we found an average 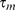 = 2.3±0.6 ms (8 carpets), corresponding to *D* = 5±1 µm^2^/s, similar to the *D* value previously reported by us for cytoplasmic NLS-GFP engaged in trasportin binding [5]. Thus we may infer that, on the cytoplasmic side, the slower pool accounts for NLS-GFP molecules co-diffusing towards the nucleus with transport machinery components (e.g. the Importin-alpha) [33]. Specularly, on the nuclear side the slower pool could account for NLS-GFPs exiting the nuclear pore but still interacting with the transport machinery, and therefore retaining the same size and diffusion coefficient. We should note that our data show the persistence of the slow NLS-GFP fraction at distances up to 500–700 nm (10–14 pixels) from the NE. Additionally, in half of the carpets, the slow-diffusing NLS-GFP pool appeared on both sides of NE regardless of the *xt* scanning direction, as showed in Fig. 5C,D. Following our hypothesis, the latter finding indicates that the complex can also travel towards the NE in the nucleus and escape from the NE in the cytoplasm. These cases could stem from complexes that either are not dissociated in the nucleus or fail to cross the NE from the cytoplasm during the observation time. Although further experiments will be required to clarify the biological significance of this phenomenon, again we should stress that it became visible only when using STED-pCF, being totally hidden in confocal mode (compare Fig. 5C,D with Fig. 7A,B).

## Conclusion

In conclusion, we here present the first demonstration of the combination of super-resolution STED imaging and the pair correlation approach to spatiotemporal correlation spectroscopy, named STED-pCF. This novel method allows monitoring intracellular protein diffusion at spatial resolution below the optical diffraction limit, we should stress that, in principle, the spatial resolution of these diffusional maps is limited only by the photophysics of the fluorescent reporter under STED conditions. To demonstrate its easy and straightforward applicability, our approach was based on the instrumentally-simple depletion by a CW source (CW-STED) on a commercial imaging system, and was applied to popular genetically-encoded fluorescent protein labels. Although CW-STED has intrinsically lower resolution as compared to pulsed depletion systems, we were able to visualize features in the 100–150 nm range, unveiling biological properties totally hidden under conventional confocal imaging. We expect these figures to be significantly improved in measurements targeting the cell membrane systems, as STED-FCS was demonstrated to give better S/N ratios when monitoring 2D diffusion, owing to the absence of low signal from axial out-of-focus areas otherwise present in 3D diffusion systems [34]. Additionally, improved super-resolution modalities such as lifetime-gated STED (g-STED) [35] have the potential to provide better spatial resolutions even using CW depletion mode. Accordingly, g-STED-pCF measurements are currently under course.

## References

[1] Hess ST, Huang S, Heikal AA, Webb WW (2002) Biological and chemical applications of fluorescence correlation spectroscopy: a review. Biochemistry 41: 697–705. 11790090 10.1021/bi0118512

[2] Digman MA, Gratton E (2009) Fluorescence correlation spectroscopy and fluorescence cross-correlation spectroscopy. Wiley Interdiscip Rev Syst Biol Med 1: 273–282. 20835996 10.1002/wsbm.5 PMC3086279

[3] Digman MA, Gratton E (2011) Lessons in fluctuation correlation spectroscopy. Ann Rev Phys Chem 62: 645–668. 21219151 10.1146/annurev-physchem-032210-103424 PMC3576135

[4] Digman MA, Brown CM, Sengupta P, Wiseman PW, Horwitz AR, et al (2005b) Measuring fast dynamics in solutions and cells with a laser scanning microscope. Biophys J 89: 1317–1327. 15908582 10.1529/biophysj.105.062836 PMC1366616

[5] Cardarelli F, Gratton E (2010) In vivo imaging of single-molecule translocation through nuclear pore complexes by pair correlation functions. PLoS ONE 5: e10475. 20454622 10.1371/journal.pone.0010475 PMC2862743

[6] Hinde E, Cardarelli F, Digman MA, Gratton E (2010) In vivo pair correlation analysis of EGFP intranuclear diffusion reveals DNA-dependent molecular flow. Proc Natl Acad Sci U S A 107: 16560–16565. 20823232 10.1073/pnas.1006731107 PMC2944750

[7] Hell SW (2009) Microscopy and its focal switch. Nat Methods 6: 24–32. 19116611 10.1038/nmeth.1291

[8] Donnert G, Keller J, Medda R, Andrei MA, Rizzoli SO, et al (2006) Macromolecular-scale resolution in biological fluorescence microscopy. Proc Natl Acad Sci U S A 103: 11440–11445. 16864773 10.1073/pnas.0604965103 PMC1518808

[9] Rankin BR, Moneron G, Wurm CA, Nelson JC, Walter A, et al (2011) Nanoscopy in a living multicellular organism expressing GFP. Biophys J 100: L63–65. 21689517 10.1016/j.bpj.2011.05.020 PMC3123922

[10] Moneron G, Medda R, Hein B, Giske A, Westphal V, et al (2010) Fast STED microscopy with continuous wave fiber lasers. Opt Express 18: 1302–1309. 20173956 10.1364/OE.18.001302

[11] Wessels JT, Yamauchi K, Hoffman RM, Wouters FS (2010) Advances in cellular, subcellular, and nanoscale imaging in vitro and in vivo. Cytometry Part A : the journal of the International Society for Analytical Cytology 77: 667–676. 20564541 10.1002/cyto.a.20931

[12] Eggeling C, Ringemann C, Medda R, Schwarzmann G, Sandhoff K, et al (2009) Direct observation of the nanoscale dynamics of membrane lipids in a living cell. Nature 457: 1159–1162. 19098897 10.1038/nature07596

[13] Mueller V, Ringemann C, Honigmann A, Schwarzmann G, Medda R, et al (2011) STED nanoscopy reveals molecular details of cholesterol- and cytoskeleton-modulated lipid interactions in living cells. Biophys J 101: 1651–1660. 21961591 10.1016/j.bpj.2011.09.006 PMC3183802

[14] Hedde PN, Dorlich RM, Blomley R, Gradl D, Oppong E, et al (2013) Stimulated emission depletion-based raster image correlation spectroscopy reveals biomolecular dynamics in live cells. Nat Comm 4: 2093. 10.1038/ncomms3093 23803641

[15] Vogel M, Diez M, Eisfeld J, Nassal M (2005) In vitro assembly of mosaic hepatitis B virus capsid-like particles (CLPs): rescue into CLPs of assembly-deficient core protein fusions and FRET-suited CLPs. FEBS letters 579: 5211–5216. 16162343 10.1016/j.febslet.2005.08.044

[16] Cardarelli F, Serresi M, Bizzarri R, Giacca M, Beltram F (2007) In Vivo Study of HIV-1 Tat Arginine-rich Motif Unveils Its Transport Properties. Mol Ther 15: 1313–1322. 17505482 10.1038/sj.mt.6300172

[17] Linkert M, Rueden CT, Allan C, Burel JM, Moore W, et al (2010) Metadata matters: access to image data in the real world. J Cell Biol 189: 777–782. 20513764 10.1083/jcb.201004104 PMC2878938

[18] Digman MA, Brown CM, Horwitz AR, Mantulin WW, Gratton E (2008) Paxillin dynamics measured during adhesion assembly and disassembly by correlation spectroscopy. Biophys J 94: 2819–2831. 17993500 10.1529/biophysj.107.104984 PMC2267137

[19] Brinkmeier M, Dorre K, Stephan J, Eigen M (1999) Two beam cross correlation: A method to characterize transport phenomena in micrometer-sized structures. Anal Chem 71: 609–616. 21662718 10.1021/ac980820i

[20] Weidemann T, Wachsmuth M, Tewes M, Rippe K, Langowski J (2002) Analysis of ligand binding by two-colour fluorescence cross-correlation spectroscopy. Single Molecules 3: 49–61.

[21] Ries J, Schwille P (2006) Studying slow membrane dynamics with continuous wave scanning fluorescence correlation spectroscopy. Biophys J 91: 1915–1924. 16782786 10.1529/biophysj.106.082297 PMC1544284

[22] Kratz PA, Bottcher B, Nassal M (1999) Native display of complete foreign protein domains on the surface of hepatitis B virus capsids. Proc Natl Acad Sci U S A 96: 1915–1920. 10051569 10.1073/pnas.96.5.1915 PMC26711

[23] Vogel M, Vorreiter J, Nassal M (2005) Quaternary structure is critical for protein display on capsid-like particles (CLPs): efficient generation of hepatitis B virus CLPs presenting monomeric but not dimeric and tetrameric fluorescent proteins. Proteins 58: 478–488. 15526302 10.1002/prot.20312

[24] Digman MA, Gratton E (2009) Imaging barriers to diffusion by pair correlation functions. Biophys J 97: 665–673. 19619481 10.1016/j.bpj.2009.04.048 PMC2711318

[25] Hinde E, Cardarelli F, Digman MA, Gratton E (2011) The Impact of Nuclear Architecture on EGFP Diffusion Revealed by Pair Correlation Analysis. Biophys J 100: 67–67. 10.1016/j.bpj.2011.02.024 PMC3072664 21463597

[26] Leutenegger M, Eggeling C, Hell SW (2010) Analytical description of STED microscopy performance. Opt Express 18: 26417–26429. 21164992 10.1364/OE.18.026417

[27] Hotta J, Fron E, Dedecker P, Janssen KP, Li C, et al (2010) Spectroscopic rationale for efficient stimulated-emission depletion microscopy fluorophores. J Am Chem Soc 132: 5021–5023. 20307062 10.1021/ja100079w

[28] Cardarelli F, Serresi M, Bizzarri R, Beltram F (2008) Tuning the transport properties of HIV-1 Tat arginine-rich motif in living cells. Traffic 9: 528–539. 18182009 10.1111/j.1600-0854.2007.00696.x

[29] Cardarelli F, Bizzarri R, Serresi M, Albertazzi L, Beltram F (2009) Probing nuclear localization signal-importin alpha binding equilibria in living cells. J Biol Chem 284: 36638–36646. 19858191 10.1074/jbc.M109.036699 PMC2794778

[30] Bizzarri R, Cardarelli F, Serresi M, Beltram F (2012) Fluorescence recovery after photobleaching reveals the biochemistry of nucleocytoplasmic exchange. Anal Bioanal Chem 403: 2339–2351. 22585053 10.1007/s00216-012-6025-4

[31] Stewart CL, Roux KJ, Burke B (2007) Blurring the boundary: the nuclear envelope extends its reach. Science 318: 1408–1412. 18048680 10.1126/science.1142034

[32] Cardarelli F, Lanzano L, Gratton E (2012) Capturing directed molecular motion in the nuclear pore complex of live cells. Proc Natl Acad Sci U S A 109: 9863–9868. 22665783 10.1073/pnas.1200486109 PMC3382504

[33] Terry LJ, Shows EB, Wente SR (2007) Crossing the nuclear envelope: hierarchical regulation of nucleocytoplasmic transport. Science 318: 1412–1416. 18048681 10.1126/science.1142204

[34] Ringemann C, Harke B, von Middendorff C, Medda R, Honigmann A, et al (2009) Exploring single-molecule dynamics with fluorescence nanoscopy. New J Phys 11.

[35] Vicidomini G, Moneron G, Han KY, Westphal V, Ta H, et al (2011) Sharper low-power STED nanoscopy by time gating. Nat Methods 8: 571–573. 21642963 10.1038/nmeth.1624

